# A Review of the Influence of Copper Slag on the Properties of Cement-Based Materials

**DOI:** 10.3390/ma15238594

**Published:** 2022-12-02

**Authors:** Qiliang Jin, Lihua Chen

**Affiliations:** 1 College of Civil Engineering, Hefei University of Technology, Hefei 230009, China; 2 Anhui Key Laboratory of Civil Engineering Structures and Materials, Hefei University of Technology, Hefei 230009, China

**Keywords:** copper slag, working properties, mechanical properties, durability, leaching properties, cement-based materials

## Abstract

Global copper slag (CS) emissions reached 57.2 million tons in 2021. Despite the increasing reuse of CS, the treatment of CS is still dominated by landfill so far, which not only occupies land resources but also causes damage to the environment. The application of CS to cement-based materials (CBMs) is one of the main approaches to its comprehensive utilization and has important economic and social implications. This article reviews the physicochemical properties, activity excitation, and heavy metal leaching properties of CS and summarizes the effect of CS on the working properties, mechanical properties, and durability of CBMs. At the end of the article, the existing problems in the research are analyzed, and the development trend is proposed, which provides technical guidance and reference for further research and application of CS in CBMs in the future.

## 1. Introduction

Copper slag (CS) is a by-product of the copper smelting process and is one of the nonferrous metal residues. CS is mainly composed of Fe_2_O_3_, SiO_2_, and a small number of metallic elements [1,2,3]. CS holds great promise for resource utilization of building materials, particularly in cement-based materials (CBMs), which can provide new ideas for overcoming the inherent shortcomings of CBMs [4,5,6,7].

In CBMs, CS can be used as supplementary cementitious materials (SCMs) to reduce cement consumption, thus saving a lot of energy consumption when producing cement [8,9]. When CS after grinding as a kind of SCMs is added to CBMs, it delays the setting time of CBMs [10,11,12,13], it does not affect the working properties of CBMs [14,15], it improves the mechanical properties of CBMs [10,16,17], and it enhances the durability of CBMs [15,18,19,20,21]. Larger particle size CS can replace the fine aggregate and coarse aggregate in some CBMs to address the resource crisis of natural sand and stone shortage. When CS used as fine aggregate is added to CBMs, it delays the setting time of CBMs [22,23,24], it enhances the workability of CBMs [25,26,27,28,29,30], it improves the mechanical properties of CBMs [30,31,32,33,34,35,36], and it enhances the durability of CBMs [19,25,26,37,38].

According to the mineral commodity summaries 2022 of U.S. Geological Survey, the total global production of refined copper in 2021 is approximately 26 million tons [39]. Production of 1 ton copper produces almost 2.2 tons of CS by-product [1], so global CS emissions reached 57.2 million tons only in 2021. Dumping or disposing of such large amounts of CS can cause space problems [30]. Moreover, toxic and harmful elements such as arsenic and lead in the CS also enter surface water system and soil with the erosion of rainwater and surface runoff, thus polluting water body and soil [40,41]. The development of the application of CS in CBMs can not only alleviate the huge problem of resource consumption but also reduce the impact of wastes on the environment, which is of great significance for economic development and environmental protection [8,26].

The overall investigations indicate that the CS has the credibility to be partially utilized in CBMs, either as SCMs, as coarse aggregates, or as fine aggregates. Some studies concluded contradictory results because of the various CS types, preparation procedures, fineness, and contents, so it is important to establish appropriate assessment standards for the composition, quality, and fineness of CS. In addition, the whole life-cycle cost of CBMs with CS and their environmental impact should be further evaluated and investigated.

## 2. Physicochemical Properties

**Figure 1:**
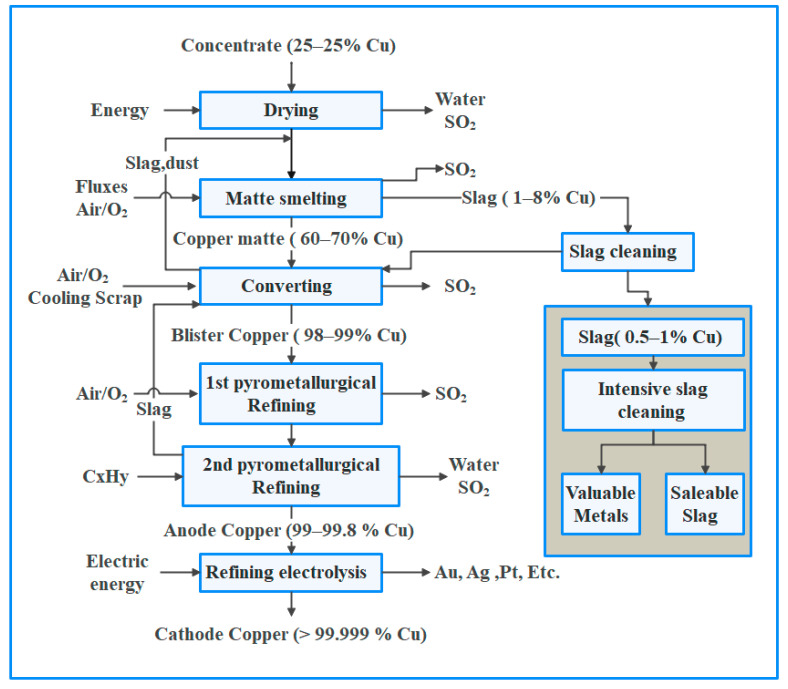
Pyrometallurgical copper extraction flow chart [46].

**Figure 2:**
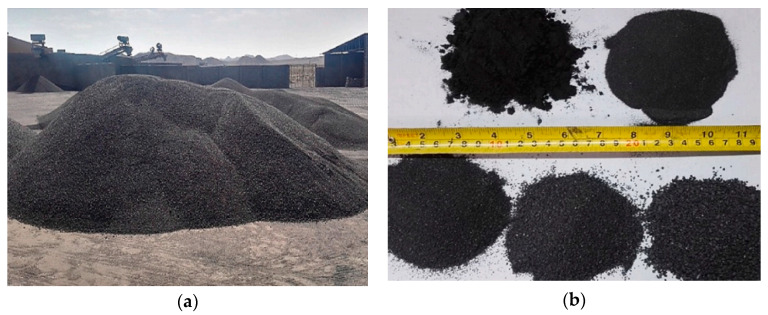
Pictures of CS: (**a**) before grinding; (**b**) after grinding [47].

**Figure 3:**
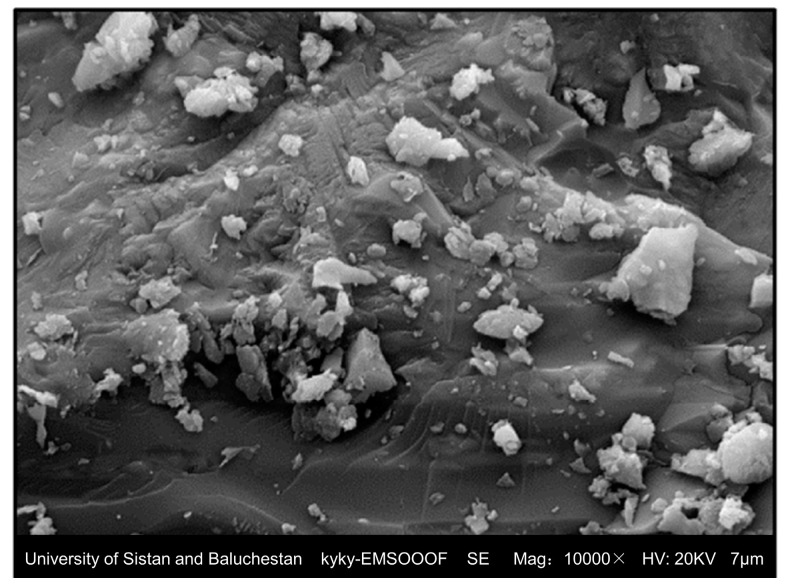
SEM morphology of CS [47].

CS is a smooth glassy by-product formed during copper pyrometallurgy (Figure 1). Depending on the cooling pattern, CS can be divided into natural cooling slag and water-quenched cooling slag. Due to different processes, CS can differ in appearance, form, and density. Naturally cooled CS is black, a glassy appearance, and most of it is dense and lumpy, brittle, and hard. The density of CS varies as the iron content changes, typically from 2.8 to 3.8 g/cm^3^. The water quenching slag cooled by water flushing at high temperature is small black particles and porous, and a small part of it is flaky and needle-like. Its packing density is 1.6–2.3 g/cm^3^ [34,42,43]. Figure 2 is a physical view of CS before and after grinding. Figure 3 is the SEM morphology of CS. Owning low water absorption of CS, it is quite obvious that CS will observe more water when compared to quartz sand [31]. CS requires less water than sand in CBMs. The wearing performs of CS is well, and the wearing-coefficient is about twice as high as that of standard sand [44]. The bulk density of CS is directly proportional to the content of Fe_2_O_3_, with a correlation coefficient of 0.94 [45]. 

**Figure 4:**
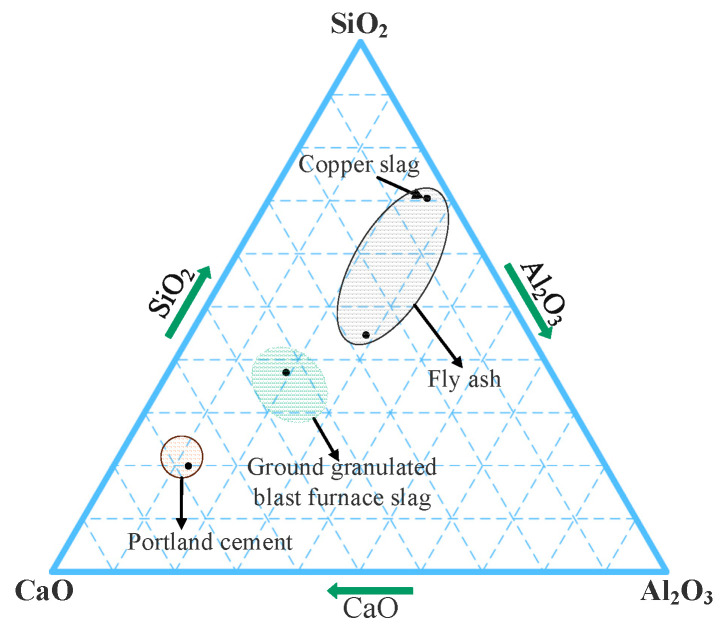
CaO-SiO_2_-Al_2_O_3_ ternary diagram (wt%) [50].

CS is mainly composed of Fe_2_O_3_, SiO_2_, and a small number of metallic elements such as calcium, magnesium, aluminum, copper, and harmful elements such as sulfur [1,2,3]. The chemical composition of typical CS in different regions is shown in Table 1. The chemical composition of activated CS is close to the slag composition [48,49]. Figure 4 is the chemical compositions of original CS in the CaO-SiO_2_-Al_2_O_3_ system [50]. CS is an amorphous vitreous body composed of fayalite (2FeO·SiO_2_), magnetite (Fe_3_O_4_), sulfide, and some gangue components [8,39,51,52]. Through SEM-EDS-MLA combined analysis, Sarfo et al. [53] found that the contents of Fe_1.2_Ca_0.5_Al_0.3_SiO_4_ and Fe_2_SiO_4_ in CS were as high as 84%, followed by Fe_3_O_4_ accounting for 11.41%, and sulfide accounted for less.

## 3. Activity Excitation

### 3.1. Physical Excitation

**Figure 5:**
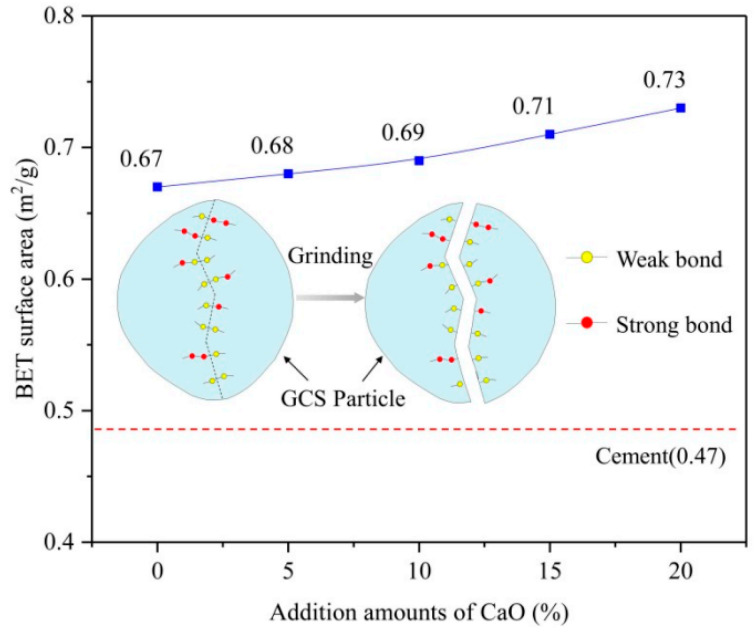
Weak bonds selective breaking in particles of GCS during grinding and GCS BET surface area of as a function of the addition amount of CaO [50].

Due to filler effect, hydration rates of CS can be accelerated by grinding, and reducing CS particle size by grinding improves the hydration of CS in CBMs [22,56,57,58]. CS is fully hydrated only if it reaches a certain fineness, CS particles bigger than 60 μm named inert particles have no positive impact on the development of strength, CS particles below 30 μm play a dominant role in the hydration, and CS particles smaller than 10 μm can be quickly hydrated, which is beneficial to early strength [59]. Nevertheless, if the CS particles are too tiny, the hydration reactants prematurely form a dense hydration product layer on the slag particles surface, affecting the formation of hydration products at later stages and affecting the strength gain at later stages [59,60]. Moreover, the cost of physical excitation increases as grinding time increases. Grinding CS for 60 min or the specific surface area of CS reaching 520 m^2^kg^−1^ is a reasonable value based on strength characteristics and manufacturing efficiency [59]. Moreover, the grindability of CS can be enhanced by adding CaO, as shown in Figure 5.

### 3.2. Chemical Excitation

**Figure 6:**
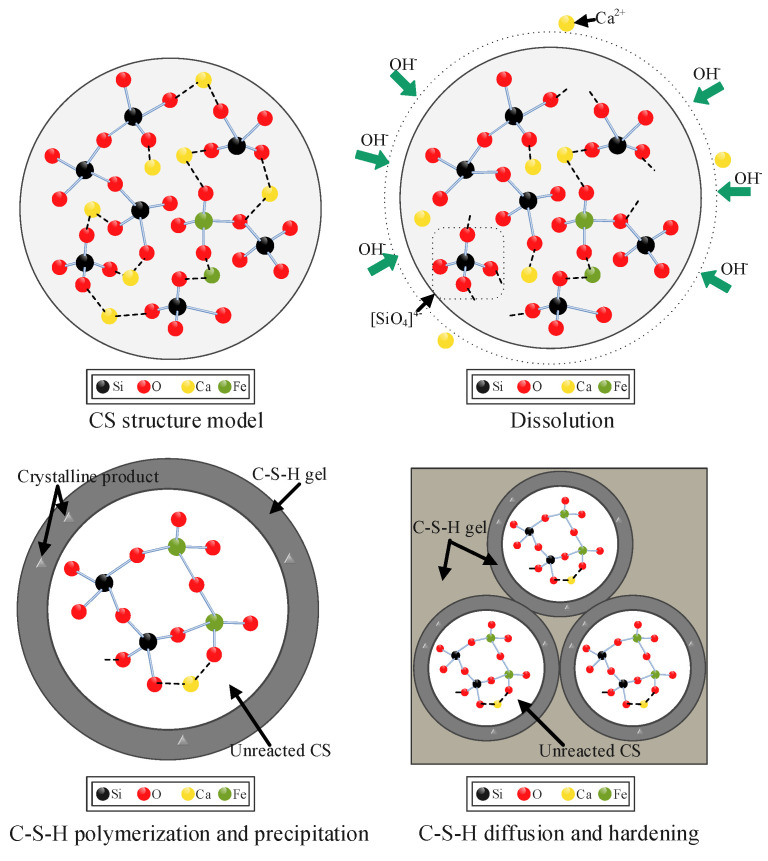
The reaction mechanism of CS with alkali activator [63].

**Figure 7:**
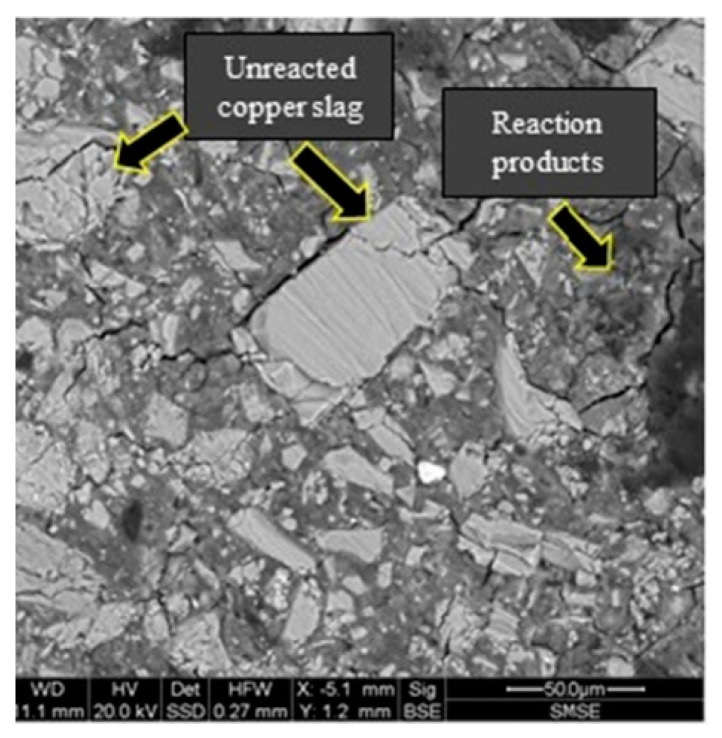
Backscattered electron image of CS activated with Na_2_O and SiO_2_ [63].

CS contains non-qualitative A1_2_O_3_ and SiO_2_, indicating that CS has volcanic ash activity. However, the fayalite, which is the main mineral component of CS, does not possess volcanic ash activity, so CS is a non-traditional volcanic ash material with low early volcanic ash activity [48,49,61]. To better exert the volcanic activity of the grinding CS, chemical activator could be added. When CS is incorporated into CBMs as SCMs, the acidic dense layer formed on the surface of CS prevents its internal substances from binding to water. When the alkali activator is introduced, the acidic layer can be destroyed. At the same time, the O-Si-O-Al-O irregular locking chain structure in CS is interrupted. Active ions in SiO_2_, CaO, MgO, Al_2_O_3_, and other components dissolve outwards, prompting the smooth progress of the secondary volcanic ash reaction. Moreover, cementitious materials with strength are generated, such as hydrated calcium silicate (C-S-H) gel, so that the strength of CBMs is increased [62]. Figure 6 shows the reaction mechanism of CS with alkali activator. Figure 7 is the backscattered electron image of CS activated with alkali activator. The unreacted CS is bright, the cracks (or pores) appear black, and the reaction products appear gray.

Lan et al. [59] activated the activity of CS by adding NaOH, triethanolamine, and lime, and found that the lime had the best excitation effect. Yan et al. [63] used sodium hydroxide and sodium silicate to develop alkali-activated CS cementitious materials, the results showed that sodium silicate had better excitation effect. The compressive strength of mortar prepared with sodium silicate in the test was six times at least higher than that of mortar prepared with sodium hydroxide. Some scholars [62] reported Ca(OH)_2_ and Na_2_SO_4_ as activator had good compatibility with CS, and the excitation effect of CS showed a trend of increase–decrease–increase, and there was an optimal mixing content of 2%. However, Na_2_SiO_3_ showed a sharp decreasing trend of excitation effect of CS, which was incompatible with CS. Song et al. [64] also explored the excitation effect of Ca(OH)_2_ and Na_2_SO_4_ on CS. Test results showed that with the increase in CS content, the activator has a more significant excitation effect on CS in CBMs. Wang [65] researched the activity excitation effect of anhydrite on CS. The results showed that anhydrite promoted the hydration reaction of CBMs more significantly at higher CS content. Wang [66] systematically studied the excitation effect and mechanism of Ca(OH)_2_ in CS cement system. The experiment showed that mixing of Ca(OH)_2_ was beneficial to improve the carbonation resistance and sulfate corrosion resistance of composite cement mortar, but it could not obviously improve the fluidity of CS cement system.

## 4. Fresh Properties

### 4.1. Setting Time

**Figure 8:**
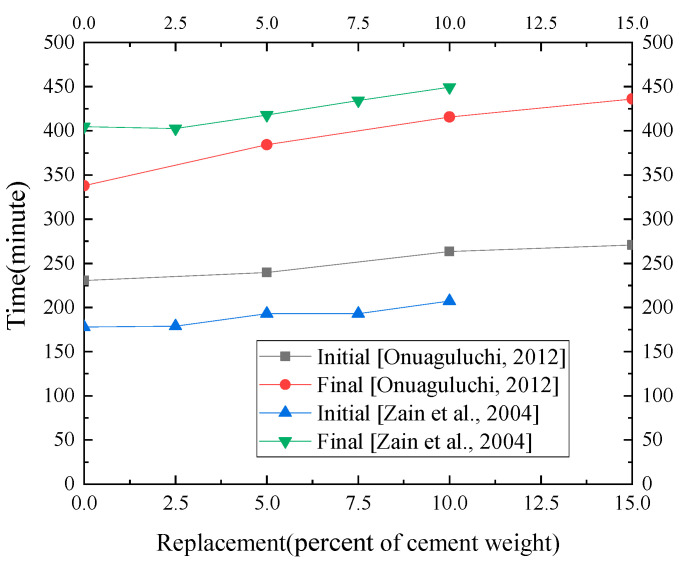
The setting time of CBMs with different amounts of CS [11,13].

When CS is used as SCMs, it delays the setting time of CBMs [10,11,12,13]. Figure 8 shows the initial setting time and final setting time of CBMs after CS displacing different proportions of cementitious materials. It can be observed that with the increasing of CS content, the setting time also increasing. When CS is used as a fine aggregate, the effect of CS of different particle sizes on the setting time of CBMs is different [8]. Ayano et al. [22] considered that the smaller the particle size of CS, the longer the setting time delay. However, the effect of CS on setting time can be reduced by washing insoluble residues on the CS surface, and the more times of washing, the smaller the effect. Resende et al. [23] found that when CS acts as fine aggregate to replace sand in mortar almost double delays the initial setting time and final setting time with the CS content ratio increasing. Lye et al. [24] concluded that the delayed setting time properties of CS in CBMs are favorable for concrete pouring in hot weather.

The reason for the delay in setting time is that CS often contains a small number of heavy metals such as Zn and Cu [10,11,15,24,67]. Studies [54,68,69,70,71] considered that Zn(OH)_2_ in CS attached to the surface of cement particles, hindering the effect of cement particles in contact with water. At the same time, Zn(OH)_2_ further reacts with C_3_S to generate CaO(Zn(OH)_2_)·2H_2_O covering the surface of C_3_S, thereby blocking the early hydration of C_3_S. Zn and hydration products generate calcium-zinc hydrate precipitation with strong retarding effect. Research [14,72,73,74,75] found that a small amount of CuO can improve the activity of C_3_S hydration and promote the formation of ettringite crystals. However, precipitation of Cu^2+^ and CuO under alkaline conditions prevents the contact of cement particles with water. The setting time of CBMs mixed with CS can be greatly shortened by adding an admixture [17,66].

### 4.2. Working Properties

Most studies have concluded that CS as SCMs does not affect the working properties of CBMs. Research [14,15] showed that when the CS substitution rate was less than 15%, CS had slight effect on the slump of concrete mixtures. Wang [66] considered that when CS was substituted for 30% of cement, the fluidity of the group mixed with CS was slightly higher than that of the cement-only control group at the initial stage, and was the same as that of the control group after 1 h. Edwin et al. [10] found that CS was not a good absorbent material, and the increase in CS as SCMs improved the workability of fresh mortar.

**Figure 9:**
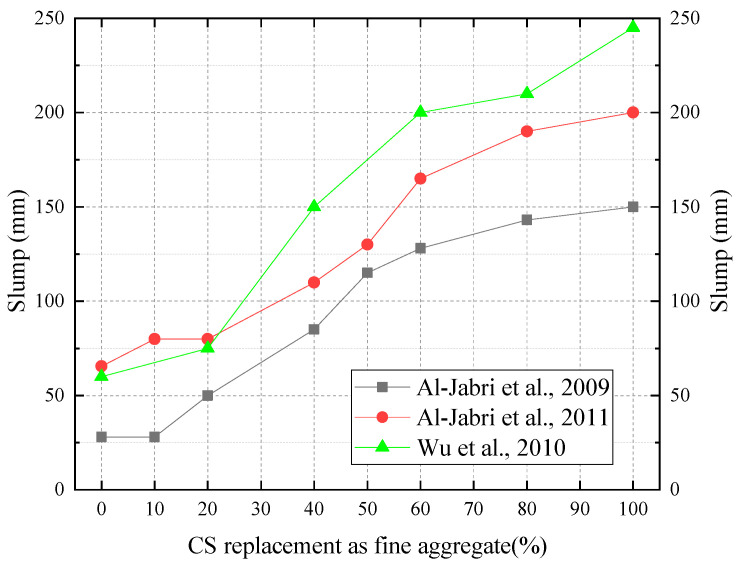
Slump of CBMs incorporated with CS replacing fine aggregate of different proportions [25,26,77].

Most studies suggest that when CS replaces fine aggregate or coarse aggregate, a certain amount of CS can enhance the workability of CBMs, but excessive CS can cause bleeding [27,28,30]. As shown in Figure 9, slump of fresh CBMs significantly increased with the increase in CS content. This is attributed to CS surface being much smoother than sand, thereby improving fluidity of CBMs. In addition, the glassy surface of CS showed lower absorption, which led to more free water can act as a lubricant between solid particles [76,77]. It should be noted that when CS content is higher than 80%, fresh CBMs show bleeding and segregation phenomena, which are detrimental to the properties of CBMs [25,26]. Shoya et al. [29] and Kumar B et al. [76] suggested the sand replacement rate of CS to be less than 40% to control the bleeding rate of fresh CBMs. In contrast to the above, Resende et al. [23] concluded that replacing the sand with CS reduced the collapse degree of the mixture, although slump remained within a reasonable range.

### 4.3. Bulk Weight

Studies [14,15] reported that the addition of CS as SCMs within 15% had little effect on the bulk weight of CBMs. Density generally increases slightly when CS is used as fine aggregate in CBMs. Research [25,26] found that the density of concrete increased by about 5% after sand was completely replaced by CS, which was chiefly due to the higher proportion of CS.

## 5. Mechanical Properties

### 5.1. Compressive Strength

Table 2 summarizes the impact of CS on the compressive strength of CBMs with different replacement patterns.

**Figure 10:**
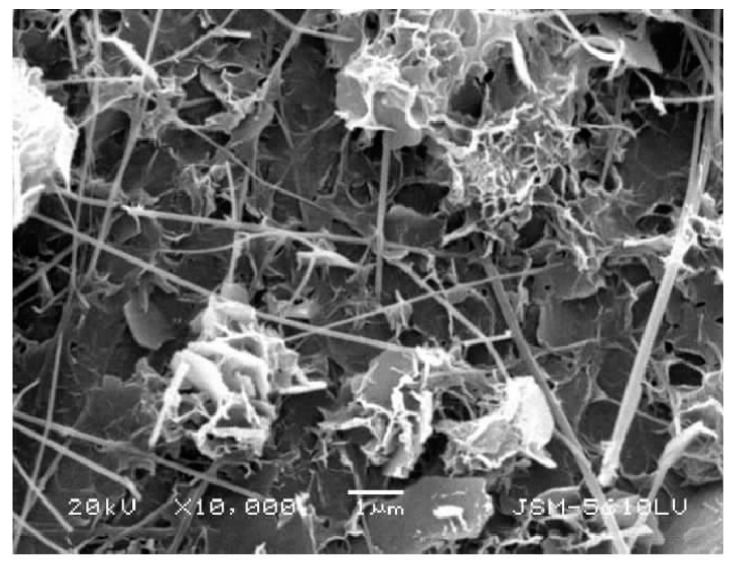
ESEM of cement paste containing CS [43].

When CS is used as SCMs, CS in most cases causes a decrease in the early compressive strength of CBMs, but the adverse effect of CS on compressive strength gradually diminishes with age, and this adverse effect can be mitigated by the addition of activator [16,17]. Active components such as silicate and aluminate can also be exposed by grinding to destroy the vitreous in CS to accelerate the early hydration reaction, thereby improving the early strength of CBMs [10]. The addition of CS allows the gap of CBMs to be filled to form a dense network structure, which is beneficial to improve the strength, as shown in Figure 10. However, when CS is used as SCMs, too high content of CS reduces the compressive strength of CBMs, and there is an optimal content. Bharath et al. [56] found that the concrete compressive strength was slightly increased when CS was substituted for 15% cement, and the compressive strength of the remaining CS mixture ratio study groups was lower than that of the 100% cement reference group. Tixier et al. [16] and Mobasher et al. [17] drew that replacing 15% cement with CS and adding 1.5% slaked lime as an activator improved the compressive strength of concrete most significantly. Edwin et al. [10] concluded that in mortar replacing cement with 10% CS was a more appropriate ratio to improve the strength of mortar. If CS is present in too high amounts, it delays the hydration reaction, which is due to limited volcanic ash activity of CS and heavy metal compounds such as zinc in CS. In addition, different water–cement ratios can affect the compressive strength of CBMs of CS with the same amount. Moura et al. [18] concluded that the 28-day compressive strength of concrete with 20% CS content was improved by 2.3%, 23.8%, and 30.4% with water–cement ratios of 0.4, 0.5, and 0.6, respectively.

As a substitute for fine aggregate, CS can enhance the CBMs compressive strength. This is due to the sharp edges of CS particles can enhance the cement matrix cohesion [33,77]. However, replacement of too much fine aggregate with CS also leads to a decrease in the CBMs compressive strength. Patil et al. [31] found that when CS substitution rate for fine aggregate was higher than 80%, concrete mixed with CS had lower strength than the pure sand concrete control group. Pavan Kumarand and Mahesh [32] drew that replacing 30% fine aggregate with CS increased the compressive strength of concrete most significantly. Chavan and Kulkarni [78] considered that the CS ratio, which increased the compressive strength most significantly, appeared at 40%. When the content of CS exceeded 40%, the concrete compressive strength began to decline after hardening. A similar phenomenon is observed in high-performance concrete. Ambily et al. [34] and Chithra et al. [35] explored the technical feasibility of using CS as a fine aggregate substitute in high-strength concrete with compressive strength greater than 150 MPa. Research found that the compressive strength of high-performance concrete increased most obviously after replacing 40% fine aggregate with CS and began to decrease after replacing more than 40%. Other studies [25,79] yielded similar results and concluded that high-performance concrete could obtain the best compressive strength when CS was used to replace 40–50% fine aggregate, and exceeding this percentage was detrimental to compressive strength. The reason for this phenomenon is that the CS low water absorption leaves too much water in CBMs. When CS is present in high amounts, CBMs produce bleeding, which leads to the formation of capillary channels and internal voids in CBMs, resulting in reduction of the compressive capacity of CBMs after hardening [33,77]. Al-Jabri [26] concluded that replacing 50% of fine aggregate with CS increased the compressive strength of mortar by more than 70%. However, the compressive strength of concrete only increased by 4.4%. The difference in strength enhancement between concrete and mortar may be due to introduction of coarse aggregate. Resende et al. [23] drew the opposite conclusion that CS replacement of sand reduced the compressive strength of mortar, although it remained within a reasonable range.

**Figure 11:**
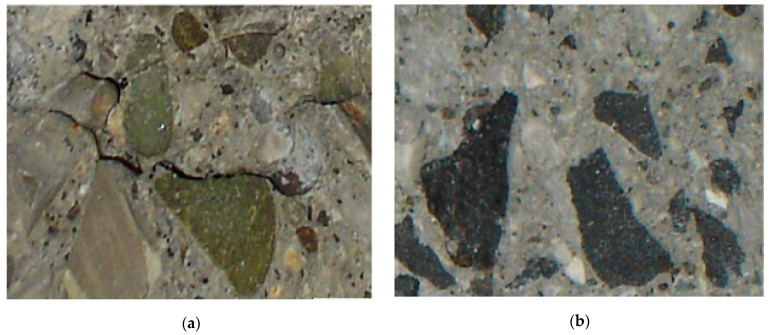
Failure sections of concrete with different coarse aggregates: (**a**) limestone coarse aggregate; (**b**) CS coarse aggregate [30].

CS may also serve as a coarse aggregate to improve the compressive strength of CBMs. Khanzadi and Behnood [30] concluded that CS incorporated into concrete as a coarse aggregate substitute for limestone improved the compressive strength of concrete by 12.9% at 91 days. This is due to the strength properties of the CS coarse aggregate. It is also attributed to the better adhesion of CS coarse aggregate and cement paste. As shown in Figure 11, CS coarse aggregate creates a better adhesive transition zone attribute to its porous and rough surface texture compared to limestone coarse aggregate.

**Table 2:** Compressive strength of CS under different replacement patterns.

Replacement Pattern	CS Replacement Ratio (%)	w/b	Type	Age (d)	Activator/ Dosage	Change Range Compared with Control Group (%)	The Optimum Dosage (%)	Refs.
Cement	0, 10, 15, 20, 30, by weight	0.55	NSC	7; 28; 56;		−23.0~1.6; −19.0~3.0; −20.9~3.5;	15	[56]
	0, 5, 10, 15, by weight	0.40	NM	1; 7; 28; 90;	lime/1.5%	−8.1~−2.4; −6.7~5.6; 14.2~31.8; 10.3~47.8;	15	[16]
	0, 5, 10, 15, by weight	0.40	NM	1; 7; 28; 90;	lime/1.5%	−10.3~−2.1; −13.1~4.1; 9.2~24.6; 7.4~45.0;	15	[17]
	0, 5, 10, 15, 20, by weight	0.15	UHPM	7; 28; 56; 90;		−8.5~21.5; −20.8~−13.3; −15.9~−4.1; −16.6~−2.9;	10	[10]
	0, 20, by weight	0.40 0.50 0.60	NSC	20;		2.3; 23.8; 30.4;		[18]
Fine aggregate	0, 10, 20, 30, 40, 50, 60, 80, 100, by weight	0.43	NSC	7; 28; 56;		−14.4~35.9; −15.9~30.6; −7.6~33.0;	20	[31]
	0, 10, 20, 30, 40, by weight	0.45	NSC	7; 14; 28;		3.9~17.6; 1.8~9.8; 2.1~9.5;	30	[32]
	0, 100, by weight	0.22	HSC	7; 14; 28;		−18.9; −11.6; −21.16;		[34]
	0, 10, 20, 30, 40, 50, 70, 90, 100, by weight	0.50 0.35	NSC HSC	7; 28; 7; 28;		9.0~19.3; 2.0~20.7; −18.6~1.2; −15.2~3.1;	50 50	[79]
	0, 10, 20, 30, 40, 50, 60, 75, 100, by weight	0.52	NSC	7; 28;		0.1~55.7; −16.3~41.7;	40	[78]
	0, 10, 20, 40, 50, 60, 80, 100, by weight	0.36	HSC	7; 28;		−18.0 ~3.8; −16.0 ~6.8;	40	[25]
	0, 25, 50, 75, by weight	0.50	NM	7; 28; 60; 90;		−27.6~−5.9; −16.7~−5.4; −33.0~−10.4; −19.8~−6.9;	25	[23]
Coarse aggregate	0, 100, by weight	0.4	HSC	7; 28; 56; 91;		11.0; 9.5; 10.7; 12.9;		[30]

Note: NSC is the normal-strength concrete; HSC is the high-strength concrete; NM is the normal mortar; UHPM is the ultra high-performance mortar.

### 5.2. Split Tensile Strength

**Figure 12:**
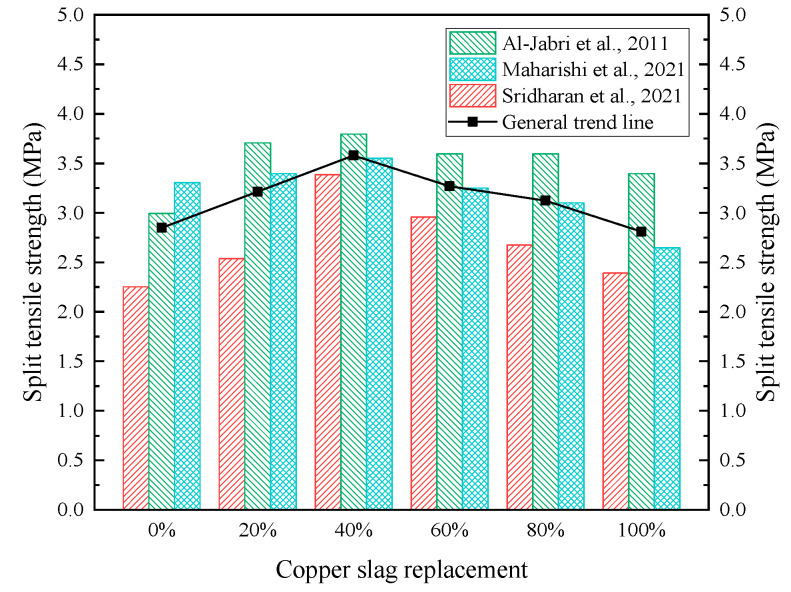
The 28-day split tensile strength of concrete incorporated with CS replacing fine aggregate of different proportions [26,46,80].

Most studies consider split tensile strength favorable when a certain amount of CS is added to CBMs as SCMs. At a water–cement ratio of 0.5, replacing 15% of cement with CS can significantly improve the split tensile strength of CBMs [18,19]. CS as fine aggregate also increases split tensile strength of CBMs [25]. It can be found from Figure 12 that the optimal content generally occurs at 40% when CS replaces fine aggregate [26,46,80]. In addition, CS can be used as a coarse aggregate to improve split tensile strength of CBMs due to the stronger binding between the CS coarse aggregate and cement paste. Split tensile strength of concrete with all coarse aggregates being CS can be more than 10% higher than that of limestone concrete [30].

### 5.3. Flexural Strength

Most studies consider CS favorable for flexural strength of CBMs, but CS exceeding a certain mixing ratio leads to a decrease in flexural strength. Optimum flexural strength of CBMs can be obtained by replacing cement with 5% CS [81,82]. In addition, replacement of 40–50% fine aggregate with CS contributes most to flexural strength of CBMs [26,80].

### 5.4. Brittleness

When different indicators are used to evaluate the effect of CS as SCMs on the brittleness of CBMs, the effect of CS is not the same, which should be given enough attention in specific occasions. Song et al. [49] used brittleness coefficient as an evaluation indicator of CS concrete brittleness, which is defined as the ratio of compressive strength to flexural strength. The larger the brittleness coefficient of concrete, the lower the toughness of concrete. The test found that the brittleness coefficient of CS concrete increased slightly compared with pure cement concrete at 7 days and 28 days, when the CS content was 5%. The brittleness coefficient of CS at 10% content was comparable to that of pure cement concrete, and the brittleness coefficient of CS concrete at 15% content was lower than that of pure cement concrete. It was concluded that CS as SCMs had the effect of reducing brittleness of concrete. Research [82] found that the uniaxial compression stress-strain curve of CS concrete was similar to that of ordinary concrete. As the proportion of CS replacing cement increased, the strength (peak value) of concrete decreased and the peak value pushed back, indicating that increasing CS content was helpful to reduce brittleness. Mobasher et al. [17] came to the opposite conclusion. Based on the three-point bending test of notched beams, critical tip opening displacement (CTODc) was used to evaluate the brittleness of CS concrete. When CS replaced 15% cement, the CTODc of concrete decreased from 0.0191 mm to 0.0081 mm, and the brittleness of CS concrete was considered to be enhanced.

## 6. Durability

### 6.1. Water Absorption

Water absorption is generally used to qualitatively evaluate the durability grade of CBMs after hardening [83,84], because concrete with poor water absorption tends to have better carbonation resistance and chloride permeation resistance [85]. Critical factors affecting water absorption are the particle size and substitution rate of CS [17,25,26].

When CS is used as SCMs, the volcanic ash activity and filler characteristics of CS contribute to the dense microstructure of concrete, which can improve the water absorption of CBMs [15]. Mobasher et al. [17] found that CS replacement of cement increased the total porosity of the sample, but most of the pores were less than 10 μm. Test showed that the decrease in the capillary porosity reduced the water absorption of CS concrete. The adverse effect of pores on CBMs after hardening is exponentially decreasing with the decrease in pore size [86]. Moura et al. [18] assessed the effect of CS as SCMs on concrete durability by capillary water absorption rate [87] and absorption test (related to total porosity) [88]. In the absorption test, produced concrete by CS replacing 20% cement reached 13.5% less water absorption than pure cement concrete, while the capillary water absorption of CS concrete decreased by 24.1%. The main reason is that CS added promotes reduction and discontinuity of capillary. Boakye [89] concluded that using 2.5%, 5%, 10%, and 15% CS as SCMs reduced the concrete water absorption from 11.5 mm/h^0.5^ to 11.0 mm/h^0.5^, 10.7 mm/h^0.5^, 10.5 mm/h^0.5^, and 10.2 mm/h^0.5^, respectively.

**Figure 13:**
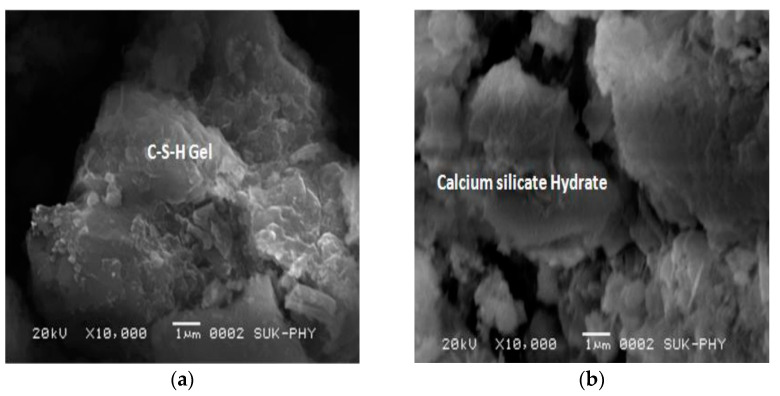

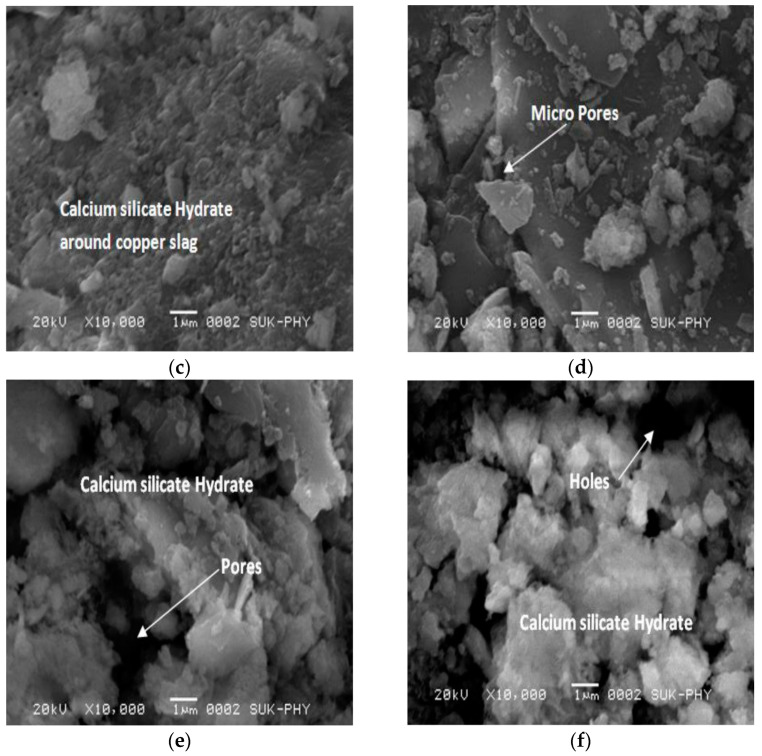
SEM micrographs of concrete with different proportions of CS at 28 days: (**a**) 0% CS + 100% sand; (**b**) 20% CS + 80% sand; (**c**) 40% CS + 60% sand; (**d**) 60% CS + 40% sand; (**e**) 80% CS + 20% sand; (**f**) 100% CS [31].

When CS replaces part of the fine aggregate, it can also improve water absorption of CBMs, but there is an optimal mixing ratio. Al-Jabri et al. [26] found mixtures that replacing fine aggregate with 40% CS demonstrated the lowest surface water absorption, which was the optimal mixing ratio. When the mixing amount of CS exceeded 40%, the concrete surface water absorption increased with increasing of CS content. Brindha et al. [37] also came to similar conclusions that substitution of sand with 40% CS decreased water absorption most significantly. As shown in Figure 13, the pores in concrete are increasing as the CS content beyond 60%, so the water absorption is enhancing, which will influence the strength of concrete with CS. Excessive CS content caused an increase in content of free water resulting in more voids in concrete; therefore, 40–50% CS is recommended as a partial replacement for fine aggregate [25,26,31,37].

### 6.2. Chloride Penetration

Because chloride penetration swells the hardened CBMs, and chloride also erodes the steel bar, improving resistance to chloride penetration is important to improve the durability of CBMs. A certain amount of CS added to CBMs helps to obtain a dense microstructure of CBMs after hardening and reduces the adverse effects of chloride penetration [20,90,91,92,93].

Most studies consider chloride penetration unfavorable when a certain amount of CS is added to CBMs as SCMs. Replacing 10% of cement with CS has the best effect on reducing chloride penetration, and the effect of CS on reducing chloride permeability is more pronounced at later stages [13,15].

**Figure 14:**
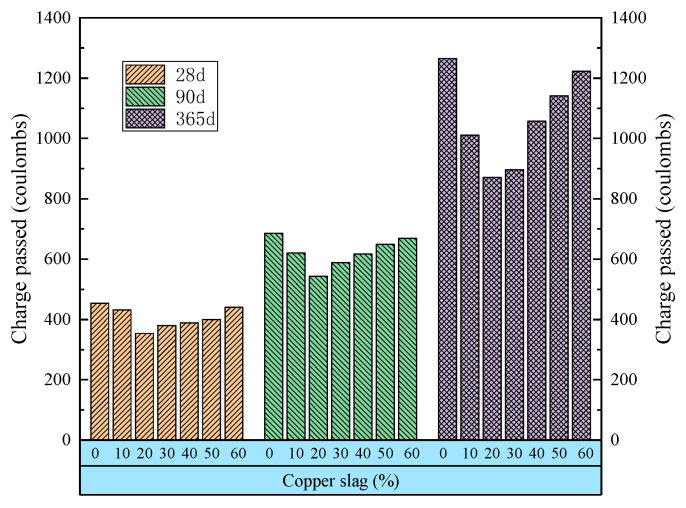
Rapid chloride penetration of self-compacting concrete with different CS ratios [94].

In terms of using CS as a fine aggregate in CBMs, replacement of 20–25% of fine aggregate with CS is the most favorable for reducing chloride penetration [69.70, 103]. Figure 14 shows that replacement of fine aggregate with 20% CS in self-compacting concrete is most effective against chloride penetration, and the effect of chloride resistance increases significantly with the increase in curing age. 

### 6.3. Carbonation

Carbonation refers to the complex physicochemical reaction between acidic gas CO_2_ and alkaline substances in CBMs, and carbonation is an important indicator in durability studies. It can be observed in Table 3 that the depth of carbonation could be reduced when CS was added to CBMs because the hardened structure of CBMs added CS was denser, limiting the diffusion rate of CO_2_ [18,19,95]. When CS was used as SCMs, the depth of carbonation decreased as the water–cement ratio decreased and decreased as the age increased [18]. When CS was used as fine aggregate, the depth of carbonation decreased as the water–cement ratio decreased [96], decreased as the coagulation time increased, and decreased as the replacement rate of CS increased [97]. Research [22,98] reported that when CS was used as part of a fine aggregate in concrete, it reduced the carbonation rate [15]. 

### 6.4. Sulfate Attack

Sulfate attack is undoubtedly one of the most serious hazards affecting the durability of CBMs [99]. The main process is that SO4^2−^ from the external environment enters the interior of CBMs and binds Ca(OH)_2_ to generate insoluble substances such as ettringite and gypsum, which cause cracking of CBMs after hardening. In addition, C-S-H decomposition is also an important reason to accelerate the destruction of hardened CBMs. Resistance to sulfate attack depends largely on the hardened CBMs permeability, and hardened CBMs with good permeability is more susceptible to sulfate attack [100,101,102,103].

**Figure 15:**
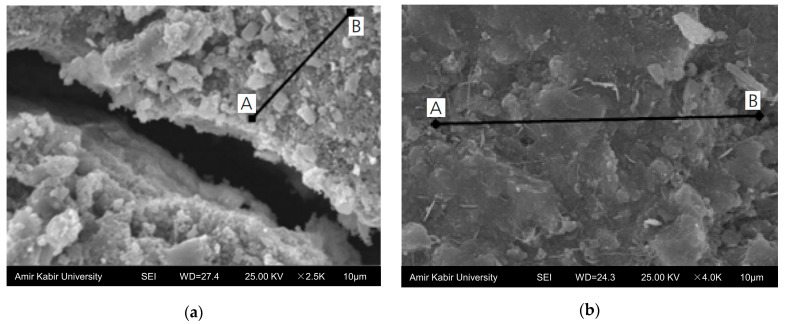
SEM diagram of CBMs mixed with CS after soaking in sulfate solution for 60 days: (**a**) 100% cement; (**b**) 10% CS + 90% cement [15].

Most studies concluded that CBMs could achieve less permeable microstructures when CS was used as SCMs. Research [14,15] reported that partial replacement of cement with CS was effective in improving CBMs resistance to sulfate solutions, and 10% CS content was a more appropriate mixing ratio. Comparing Figure 15a,b, there are cracks in SEM pictures of pure cement concrete soaked in sulfate solution for 60 days, which generally appear in calcium hydroxide (Ca(OH)_2_) crystals (CH), while no cracks appear in concrete after adding CS and the texture of concrete is denser. Wang [104] reported that the addition of CS as SCMs was advantageous against sulfate attack, with an optimal mixing ratio of 20%. It should be noted that increasing CS specific surface area against sulfate attack is advantageous at certain CS content, but it is not the larger the better. When the specific surface area of CS is too large, the active ions will dissolve too quickly after the CS is activated, and the hydration silicate gel filling of the secondary volcanic ash reaction product is not uniform, so there are different changes in sulfate attack. Sulfate resistance of CBMs mixed with CS can be effectively improved by adding appropriate amounts of activator (e.g., Ca(OH)_2_ and Na_2_SO_4_) [66,104]. CBMs also exhibit higher resistance to sulfate attack when CS replaces sand as fine aggregate [37,38]. The formation of ettringite and the precipitation of sulfate will increase the mass of CBMs. Maharishi et al. [46] found that 40% of CS replacing sand had the least concrete mass change in sulfate solution. Concrete mass increased by only 0.04% after 56 days of exposure, which was lower than that of the pure sand control group.

### 6.5. Other Durability

**Figure 16:**
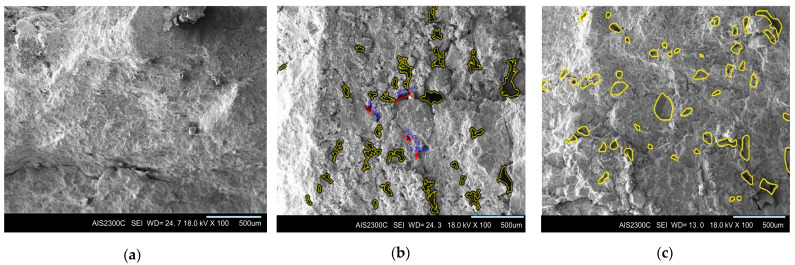
Concrete specimen SEM micrographs at different CS replacement: (**a**) 100% sand; (**b**) 40% CS + 60% sand; (**c**) 100% CS [105].

Peirovi et al. [105] reported that CS as a fine aggregate can obviously enhance the freeze–thaw resistance of CBMs. When 40% CS was used to replace sand, CS concrete mass loss was minimal under freezing and thawing conditions. As shown in Figure 16, the pore spacing and pore size on the surface of the test sample with 40% CS mixing ratio are relatively better than those of the other test samples, and yellow border is the pore location. Excessive CS can instead cause increased free water in the mixture and increased pores, resulting in reduced freeze–thaw resistance of CBMs. Afshoon and Sharifi [106] investigated the high-temperature behavior of self-compacting concrete after adding CS. When CS was used to replace 5% cement, self-compacting concrete performed best at all temperatures. Excessive CS content would adversely affect the performance of self-compacting concrete when the temperature exceeds 200 °C. Ma et al. [107] found that CS replacing cement by less than 15% did not adversely affect the mechanical properties of mortar when the temperature was lower than 200 °C.

## 7. Leaching Properties

### 7.1. Heavy Metal Leaching Toxicity of CS

Heavy metal leaching toxicity of CS in studies across countries is required to meet the respective regional safety limits. Brindha et al. [37] immersed CS in distilled water for 15 days, and no leaching of heavy metals such as lead, zinc, chromium, nickel, and molybdenum was observed. Shanmuganathan et al. [108] reported that CS would not be significantly dissolved even if it was repeatedly leached under natural acid rain, so the heavy metals in CS were very stable, and CS was safe to mix with Portland cement. Researchers [109,110,111] believed that CS was suitable for use as filling material for land reclamation, and the content of leached elements in CS at pH 5.0 was significantly lower than the limit value of TCLP [112] recommended by the US Environmental Protection Agency. Some reports [113,114,115,116,117,118] believed that CS was a potential source of heavy metal pollution of soil and water resources in surrounding areas, and CS would release heavy metals beyond the limit level when CS was exposed to acid rain.

### 7.2. Toxicity of CBMs Mixed with CS

After adding CS to CBMs to harden, due to adsorption, encapsulation and ion group replacement, the heavy metals in CS were solidified in the hardened CBMs, which greatly limited the ability of heavy metals to diffuse to the environment and essentially eliminated the ecological risks of heavy metal components [119,120,121]. Taking CS as building material will pose neither human risk nor environmental risk. Wang et al. [122] reported that the leaching concentration of harmful elements in the leaching solution of 3d and 28d CBMs blocks mixed with CS did not exceed the toxicity identification criteria limit for leaching of harmful elements in solid waste, and the concentration of harmful elements in the leaching solution was lower than the limit of Grade III water-quality standard [123,124,125]. Alp et al. [113] added CS as SCMs into mortar, and the metal release in hardened mortar was lower than the regulatory limit of TCLP [112]. Zain et al. [11] added the derusting CS with heavy metal content much higher than ordinary CS into concrete. The leaching concentration of heavy metal of derusting CS concrete was still significantly lower than the environmental standards of the United States and Malaysia. Onuaguluchi [13] found that as CS content increased, CBMs hardened to fix heavy metals to a greater extent. This phenomenon was attributed to the denser microstructure of CBMs due to the addition of CS.

## 8. Conclusions

In general, research results show that CS can enhance the mechanical properties and durability of CBMs and improve the workability of CBMs. However, the higher CS content will lead to a decrease in mechanical properties and durability of CBMs owing to low water absorption of CS. Hence, the optimum dose of CS is suggested to use. Moreover, using CS as building material is safe and can be promoted. The specific conclusions are as follows:

1. CS is a non-traditional volcanic ash material with low early activity. Adding appropriate amounts of alkali chemical activators (e.g., 2% Ca(OH)_2_) can damage the acidic dense layer on the CS surface and promote the smooth progress of the secondary volcanic ash reaction, thereby improving the performance of CBMs mixed with CS in all aspects.
2. CS contains a small number of heavy metals such as Zn and Cu, which can have an effect on the setting time of CBMs mixed with CS. The effect of CS on the setting time of SCMs can be decreased by reducing the CS particle size or washing the insoluble residues on the CS surface. In summary, the addition of CS is beneficial to improve the workability of CBMs and decrease the water demand of the mixture, but the addition of CS more than 80% will cause increased bleeding rate, so it is necessary to control the content of CS in CBMs. To improve the workability of CBMs with CS, the optimal ratio of CS to replace cementitious materials is 30%, while the optimal ratio of CS to replace the fine aggregate is 40–50%.
3. CS is beneficial for improving long-term strength of CBMs but has a weakening effect on the development of early strength, which can be mitigated by the addition of chemical activators. In terms of improving the mechanical properties of CBMs, most reports suggest that the optimal ratio of CS to replace cementitious materials is 5–20%, while the optimal ratio of CS to replace the fine aggregate is 40–50%.
4. Volcanic ash activity and filler properties of CS contribute to CBMs to a compact microstructure, thereby improving durability of CBMs. However, too much CS increases the amount of free water in CBMs, resulting in more pores after the hardening of CBMs. The ratio of CS as SCMs replacement is recommended to be limited to 20%. The substitution ratio is recommended to be limited to 40% when CS is used as fine aggregate.
5. Heavy metal leaching toxicity of CS in various countries meets the safety limits of each region, but there are also relevant reports that CS is a potential source of heavy metal pollution in soil and water in surrounding areas, so all CS cannot be regarded as harmless wastes. However, after adding CS to CBMs to harden, various heavy metals in CS are solidified in the hardened CBMs due to adsorption, encapsulation, and ion group replacement, essentially eliminating the ecological risk of heavy metal components of CS. Therefore, using CS as building material does not pose human and environmental risks.

## 9. Challenges and Prospects

The overall investigations indicate that the CS has the credibility to be partially utilized in CBMs, either as SCMs, as coarse aggregates or as fine aggregates. Some studies concluded contradictory results because of the various CS types, preparation procedures, fineness, and contents. The long-term durability of CBMs mixed with CS is still an important concern, which limits greatly its extensive application. Therefore, more attention should be considered on the following research directions:

1. It is important to establish appropriate assessment standards for the composition, quality, and fineness of CS. Criteria including mixing procedures and curing procedures should be also established for CBMs incorporated with CS to obtain optimal durable performances and mechanical properties.
2. The information on some durability for instance creep and dry shrinkage properties of CBMs with CS is still limited, and the research work in this area needs to be further improved. In addition, concrete constructions are subject to complex erosion by multiple ions in practical engineering, and the attack law of composite ions on CBMs containing CS needs to be further studied.
3. The skid resistance and abrasion of CBMs with CS is significant but investigated rarely when the CS is utilized to the highway or pavement.
4. CS can be applied in CBMs, and the mechanical properties can be improved, but CBMs is still in a low tension. Hence, further studies were proposed to improve the ductility of CBMs with the supplement of different fibers.
5. It is necessary to further study the effect of activator on corrosion resistance, freeze–thaw resistance, and carbonation resistance CBMs mixed with CS, providing scientific and technical support for the application of CS to break through regional limitations.
6. The whole life-cycle cost of CBMs with CS and their environmental impact should be further evaluated and investigated.

## Figures and Tables

**Table 1:** CS chemical composition from different regions by mass (%) [8,54,55].

Country	Fe_2_O_3_	SiO_2_	Al_2_O_3_	CaO	MgO	SO_3_	CuO
Chile	20.40	38.33	8.17	26.10	2.14	—	—
China	57.80	29.07	4.02	2.30	2.70	0.32	—
USA	44.80	24.70	15.60	10.90	1.70	0.28	2.10
Canada	49.50	34.51	6.55	2.20	1.48	1.20	0.43
Australia	45.30	36.00	3.45	9.30	3.24	0.49	0.33
Japan	52.00	35.50	5.90	2.11	1.06	0.14	0.88
Spain	60.00	30.07	3.97	0.60	0.75	0.32	0.79
Brazil	62.00	26.00	—	2.50	3.70	—	1.40

**Table 3:** Carbonation resistance of CS under different replacement patterns.

Replacement Pattern	CS Replacement Ratio (%)	w/b	Type	Age (d)	Carbonation Average Thickness Change Range Compared with Control Group (%)	The Optimum Dosage (%)	Refs.
Cement	0, 20, by weight	0.50 0.60	OPC	210; 240; 210; 240;	−100; −80; −57.1; −35.7;	20	[18]
Fine aggregate	20, 40, 60, 80, by weight	0.45 0.55	OPC	300;	−84.4~−37.5; −78.3~−43.8;	40 60	[96]
	0, 20, 40, 60, 80, 100, by weight	0.45	OPC+ MK + FA	28; 56; 84; 112;	−26.2~−3.7; −34.1~−5.6; −36.8~−3.7; −41.0~−4.2;	100	[97]

Note: OPC is the ordinary Portland cement; FA is the fly ash; MK is the metakaolin.

## Data Availability

Not applicable.
